# SARS-CoV-2–specific immune responses in boosted vaccine recipients with breakthrough infections during the Omicron variant surge

**DOI:** 10.1172/jci.insight.159474

**Published:** 2022-05-23

**Authors:** Bezawit A. Woldemeskel, Caroline C. Garliss, Tihitina Y. Aytenfisu, Trevor S. Johnston, Evan J. Beck, Arbor G. Dykema, Nicole Frumento, Desiree A. Wright, Andrew H. Yang, Alexander I. Damanakis, Oliver Laeyendecker, Andrea L. Cox, Heba H. Mostafa, Andrew H. Karaba, Joel N. Blankson

**Affiliations:** 1 Department of Medicine, Johns Hopkins School of Medicine, Baltimore, Maryland, USA.; 2 Division of Intramural Research, National Institute of Allergy and Infectious Diseases, NIH, Bethesda, Maryland, USA.; 3 The Bloomberg~Kimmel Institute for Cancer Immunotherapy, Baltimore, Maryland, USA.; 4 The Sidney Kimmel Comprehensive Cancer Center, Baltimore, Maryland, USA.; 5 Department of Pathology, The Sol Goldman Pancreatic Cancer Research Center, Johns Hopkins School of Medicine, Baltimore, Maryland, USA.; 6 Department of General, Visceral, Cancer and Transplant Surgery, University Hospital of Cologne, Cologne, Germany.; 7 Department of Pathology, Division of Medical Microbiology, Johns Hopkins School of Medicine, Baltimore, Maryland, USA.

**Keywords:** COVID-19, Adaptive immunity, T cells

## Abstract

**Background:**

Breakthrough SARS-CoV-2 infections in vaccinated individuals have been previously associated with suboptimal humoral immunity. However, less is known about breakthrough infections with the Omicron variant.

**Methods:**

We analyzed SARS-CoV-2–specific antibody and cellular responses in healthy vaccine recipients who experienced breakthrough infections a median of 50 days after receiving a booster mRNA vaccine with an ACE2 binding inhibition assay and an ELISpot assay, respectively.

**Results:**

We found that high levels of antibodies inhibited vaccine strain spike protein binding to ACE2 but that lower levels inhibited Omicron variant spike protein binding to ACE2 in 4 boosted vaccine recipients prior to infection. The levels of antibodies that inhibited vaccine strain and Omicron spike protein binding after breakthrough in 18 boosted vaccine recipients were similar to levels seen in COVID-19–negative boosted vaccine recipients. In contrast, boosted vaccine recipients had significantly stronger T cell responses to both vaccine strain and Omicron variant spike proteins at the time of breakthrough.

**Conclusion:**

Our data suggest that breakthrough infections with the Omicron variant can occur despite robust immune responses to the vaccine strain spike protein.

**Funding:**

This work was supported by the Johns Hopkins COVID-19 Vaccine-related Research Fund and by funds from the National Institute of Allergy and Infectious Disease intramural program as well as awards from the National Cancer Institute (U54CA260492) and the National Institutes of Allergy and Infectious Disease (K08AI156021 and U01AI138897).

## Introduction

The Omicron variant of concern (VOC) (B.1.1.529) was identified in November 2021 in South Africa and has since spread across the globe, replacing the Delta variant as the dominant strain (1). Omicron has over 50 mutations in its genome, with over 30 mutations residing in the spike protein (1). There is evidence that Omicron is more transmissible (1) and infectious (2) than previous VOCs. Moreover, Omicron effectively evades vaccine-elicited neutralizing antibodies, with 2 doses of COVID-19 mRNA vaccine inducing minimal antibody responses that can cross-neutralize Omicron (3–8). Booster doses enhance levels of Omicron-neutralizing antibodies; however, these responses remain 4–6 times lower than responses to vaccine strain spike protein (3–6). Unlike neutralizing antibodies, vaccine-induced T cell responses can cross-recognize the omicron spike protein (9–15), and this may partially explain protection against severe disease.

COVID-19 mRNA vaccines have strong efficacy against prior VOCs, including the Delta variant; however, the efficacy is much lower against the Omicron variant after a 2-dose COVID-19 mRNA vaccination regimen (16–19). One study found that vaccine efficacy against the Omicron variant infection was 44% at 14–90 days following the second dose and declined dramatically over time (16). A second study found vaccine effectiveness against symptomatic infection after 2 BNT162b2 doses was 65.5% at 2–4 weeks, but this dropped to 8.8% after 25 weeks (19).

A third vaccine dose increases protection from all VOCs; however, efficacy against the Omicron variant remains much lower compared with the Delta variant and declines over time. Andrews et al. reported that vaccine effectiveness against symptomatic Omicron variant infection increased to 67.2% at 2–4 weeks after a BNT162b2 booster dose, before declining to 45.7% at 10 weeks (19). In another study, Tseng et al. showed that vaccine effectiveness against infection 2 months after a booster dose was 86% against the Delta variant and 47% against the Omicron variant (16).

Breakthrough infections with the Alpha variant in fully vaccinated individuals have been associated with lower titers of neutralizing antibodies (20–22) and less robust T cell responses (23). However, given that the Omicron variant has more mutations and evades neutralizing antibody responses better than prior VOCs, the mechanisms of Omicron variant breakthrough infections are likely different. Thus, it is important to analyze immune responses prior to and following Omicron variant breakthrough infections in fully vaccinated as well as boosted individuals.

In this study, we determined antibody and T cell responses following breakthrough infections in 18 individuals who had received a booster mRNA vaccine (referred hereafter as breakthrough VRs) during the Omicron variant surge. Importantly, we were able to study immune responses in 4 breakthrough VRs prior to the occurrence of breakthrough infections. Our data advance our understanding of breakthrough infections in vaccinated individuals.

## Results

### Breakthrough VRs have high levels of vaccine strain spike-binding antibodies.

**Figure 1:**
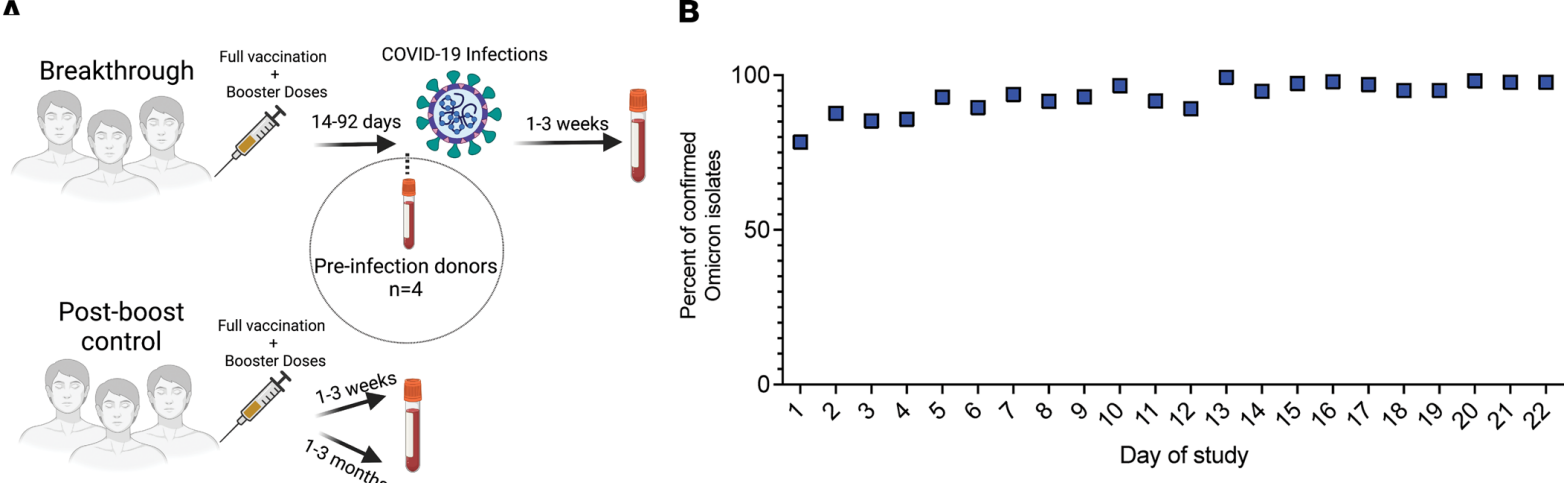
Design of observational study of boosted vaccine recipients with breakthrough infections. (**A**) Observational study design. (**B**) The frequency of Omicron cases among sequenced SARS-CoV-2 isolates at Johns Hopkins Hospital during the study period.

We tested antibody levels in 18 breakthrough VRs. Fifteen breakthrough VRs received mRNA COVID-19 vaccinations and boosters, and 3 received the Ad26.COV2 (Johnson & Johnson) vaccine followed by an mRNA vaccine booster. The median time between the receipt of the booster dose and the onset of symptoms was 50 days (range, 14–92 days). The median time between symptom onset and sampling was 11 days (range, 6–19 days). For 4 breakthrough VRs (VR21, VR26, VR37, and VR97), we analyzed immune responses between 5 and 21 days after the booster vaccine dose and prior to breakthrough infection. The study design is illustrated in Figure 1A, and information on the breakthrough VRs is presented in Table 1. While we were not able to document infection with the Omicron strain in breakthrough VRs, the participants were infected when this VOC accounted for more than 90% of the SARS-CoV-2 isolates sequenced at the Johns Hopkins Hospital during a 22-day time period in late December 2021 to mid-January 2022 when the VRs became symptomatic (ref. 24 and Figure 1B). In addition, we tested immune responses in individuals prior to receiving booster doses, more than 6 months following their second mRNA vaccine (referred here after as preboost VRs). Furthermore, we tested immune responses in individuals who had no history of COVID-19 and who received booster doses (referred to as postboost VRs), at either 1–3 weeks or 1–3 months following their booster vaccination.

**Figure 2:**
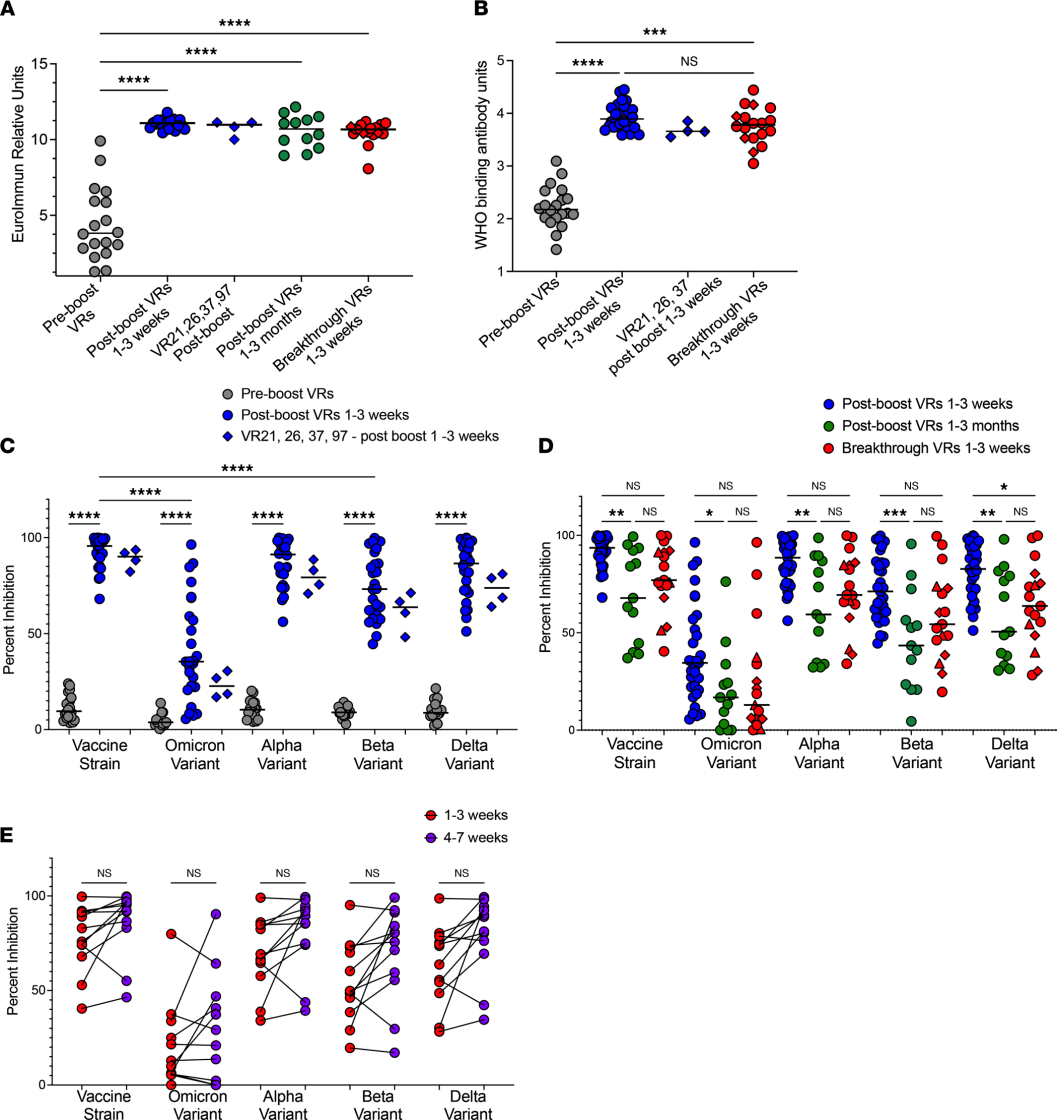
Characterization of antibody levels in boosted vaccine recipients with breakthrough infections. (**A**) Spike-binding antibodies found in fully vaccinated individuals prior to their booster shots (Pre-boost VRs), individuals 1–3 weeks (Post-boost VRs 1–3 weeks) or 1–3 months (Post-boost 1–3 months) after their booster shot, breakthrough VRs 1–3 weeks after symptom onset, and VR21, VR26, VR37 and VR97 1–3 weeks after their booster shots and before they experienced breakthrough infections. The orange diamonds represent VR21, VR26, VR37, and VR97 at the breakthrough time point. The orange triangles represent participants who received the Ad26.COV2 vaccine followed by the mRNA1273 booster vaccine. (**B**) Receptor binding domain (RBD) antibodies in preboost, postboost, and breakthrough VRs as well as VR21, VR26, and VR37 at the postboost time point and prior to infection. The orange diamonds represent VR26 and VR37 at the breakthrough time point. Data are presented in log scale. (**C**) Levels of antibodies that inhibit ACE2/spike binding in preboost and postboost VRs and in VR21, VR26, VR37, and VR97 at the 1- to 3-week postboost time point. (**D**) Levels of antibodies that inhibit ACE2/spike binding in postboost VRs 1–3 weeks or 1–3 months after their booster shot and breakthrough VRs 1–3 weeks after symptom onset The orange diamonds represent VR21, VR26, VR37, and VR97. (**E**) Levels of antibodies that inhibit ACE2/spike binding in paired breakthrough VRs at 1–3 weeks and 4–7 weeks after infection. Statistical comparisons were done using ordinary 1-way ANOVA (unpaired) and Holm-Šidák’s multiple comparison test, with a single pool variance used (**A**–**D**). (**E**) Paired analyses were performed using repeated-measures 1-way ANOVA with Geisser-Greenhouse corrections, and Šidák’s multiple comparison test, with individual variances was computed for each comparison. *P = 0.0332, **P = 0.0021, ***P = 0.0002, ****P < 0.0001.

As expected, binding antibodies against vaccine strain spike (Figure 2A) and the receptor binding domain (Figure 2B) were significantly higher in postboost VRs compared with preboost VRs, as measured by the Euroimmun (25) and Meso Scale Diagnostics (MSD) binding assays, respectively. In the 4 donors for whom we have prebreakthrough samples (VR21, VR26, VR37, and VR97), antibody levels 1–3 weeks after boosting were comparable to the postboost levels seen in postboost VRs, indicating that these breakthrough VRs had strong peak antibody responses following booster doses (Figure 2, A and B). Because postboost VRs had antibody levels tested 1–3 weeks after the booster shot, whereas breakthrough VRs were tested a median of 67 days after the booster shot (but 1–3 weeks following infection), we also compared the antibody levels from breakthrough VRs to postboost VRs 1–3 months (median of 77 days) after the booster shot (Figure 2A). The levels of spike-binding antibody were similar to the levels seen at months 1–3 in postboost VRs with the Euroimmun assay.

### Breakthrough VRs have high levels of antibodies that inhibit ACE2 binding to the vaccine strain spike protein.

We tested antibody-mediated inhibition of spike proteins binding to ACE2 using the MSD pseudoneutralization/ACE2 inhibition assay, which has been shown to correlate well with a culture-based neutralization assay (26). The degree of ACE2/spike protein binding inhibition was much higher in postboost VRs than in preboost VRs (Figure 2C). In postboost VRs, there was reduced inhibition of ACE2 binding to the spike proteins from the Omicron and Beta variants compared with the vaccine strain and a very wide range of inhibition of ACE2 binding to the Omicron spike protein. The postboost plasma from VR21, VR27, VR37, and VR97 strongly inhibited binding of ACE2 to the vaccine strain spike protein. In contrast, inhibition of ACE2 binding to the Omicron spike protein was at the lower end of the spectrum seen with plasma from postboost VRs (Figure 2C). This was also observed to a lesser extent for the Alpha, Beta, and Delta variants. Interestingly, plasma samples from breakthrough VRs inhibited binding of ACE2 to all 5 spike proteins to a degree that was similar to the level seen with plasma obtained from postboost VRs at the 1- to 3-month time point, indicating that infection with the Omicron variant did not enhance ACE2-inhibiting antibodies (Figure 2D). In order to determine the kinetics of antibody responses, we analyzed ACE2-inhibiting antibodies against different variants in breakthrough VRs 4–7 weeks following symptom onset. We found that antibody levels against all variants tested were slightly higher at this time point, but the differences were not statistically significant (Figure 2E), and there was no correlation between time after symptom onset and the levels of ACE2-inhibiting antibodies (Supplemental Figure 1; supplemental material available online with this article; https://doi.org/10.1172/jci.insight.159474DS1).

### Breakthrough VRs have robust T cell responses to the vaccine strain and Omicron variant spike protein.

**Figure 3:**
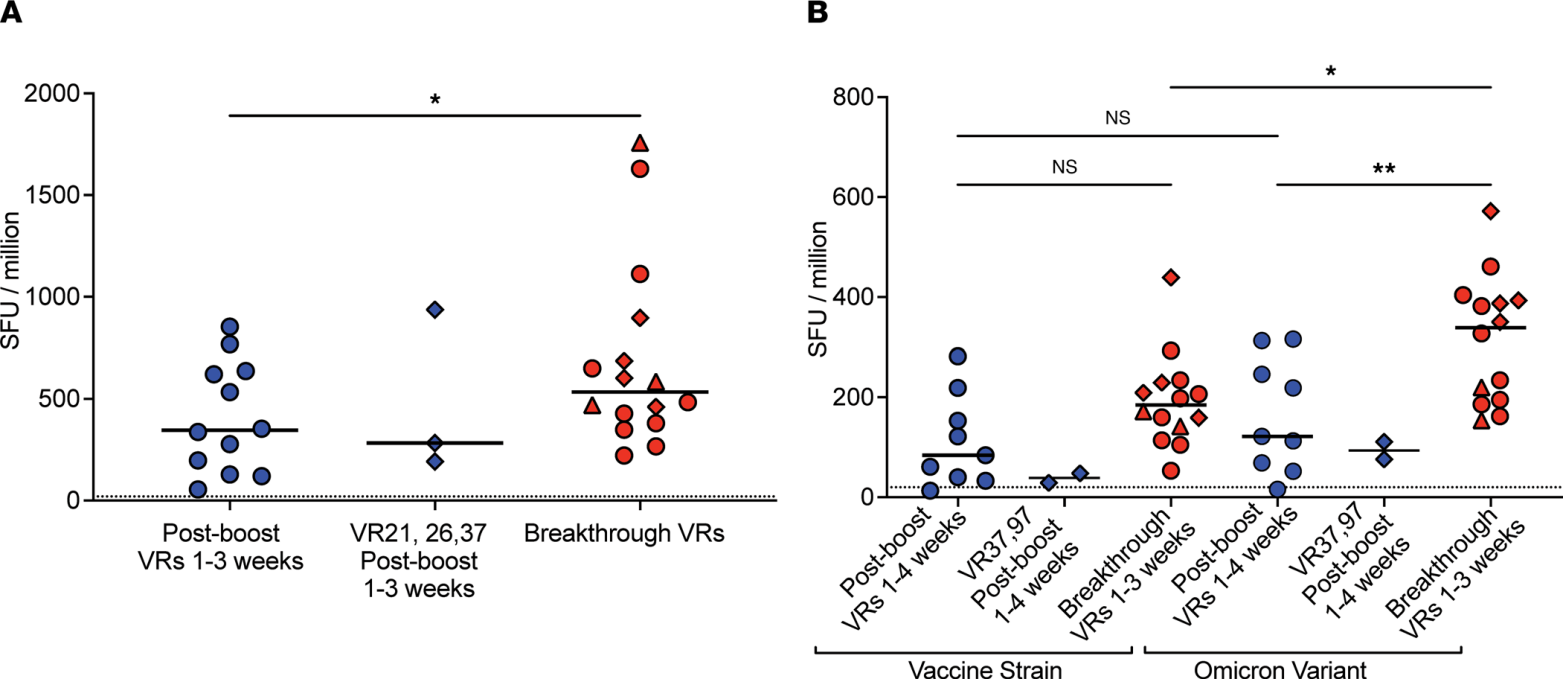
Characterization of SARS-CoV-2–specific T cells in boosted vaccine recipients with breakthrough infections. IFN-γ ELIspot assay was performed with overlapping spike peptide pools from (**A**) vaccine strain and (**B**) S1 spike proteins from vaccine strain or Omicron variant in VR21, VR26, and VR37 1–3 weeks after boost in A; in VR37 and VR97 1–3 weeks after boost in B; and in breakthrough VRs. IFN-γ spot-forming units (SFU) per million PBMCs are shown. Orange diamonds represent VR21, VR26, VR37, and VR97 at the breakthrough time point. Statistical comparisons were done using ordinary 1-way ANOVA (unpaired) and Holm-Šidák’s multiple comparison test, with a single pool variance used. *P =0.0332, **P =0.0021.

We subsequently analyzed T cell responses to the vaccine strain spike protein with the ELISpot assay using overlapping spike peptide pools (27). The number of IFN-γ spot-forming units (SFUs) measured in VR21, VR26, and VR37 at the postboost time point was similar to that of postboost VRs, indicating strong peak responses following booster doses in these 3 individuals who eventually had breakthrough infections (Figure 3A). Interestingly, PBMCs from the breakthrough VRs generated stronger responses to vaccine strain spike peptides than did PBMCs from postboost VRs (Figure 3A). We also compared responses to the vaccine strain and Omicron variant spike protein S1 subunits and found that the breakthrough VRs made similar responses to the vaccine strain but more potent responses to the Omicron S1 protein than the postboost VRs (Figure 3B), indicating that T cell responses to Omicron variant are likely enhanced by breakthrough infections to this variant.

Because all the postboost VRs received only mRNA vaccines, we asked whether inclusion of 3 breakthrough VRs who received the Ad26.COV2 vaccine followed by mRNA1273 booster shots affected our results. Exclusion of these 3 breakthrough VRs did not change the results obtained in our antibody and T cell analyses (Supplemental Figure 2).

### Longitudinal antibody and T cell responses to vaccine strain and Omicron variant spike protein.

**Figure 4:**
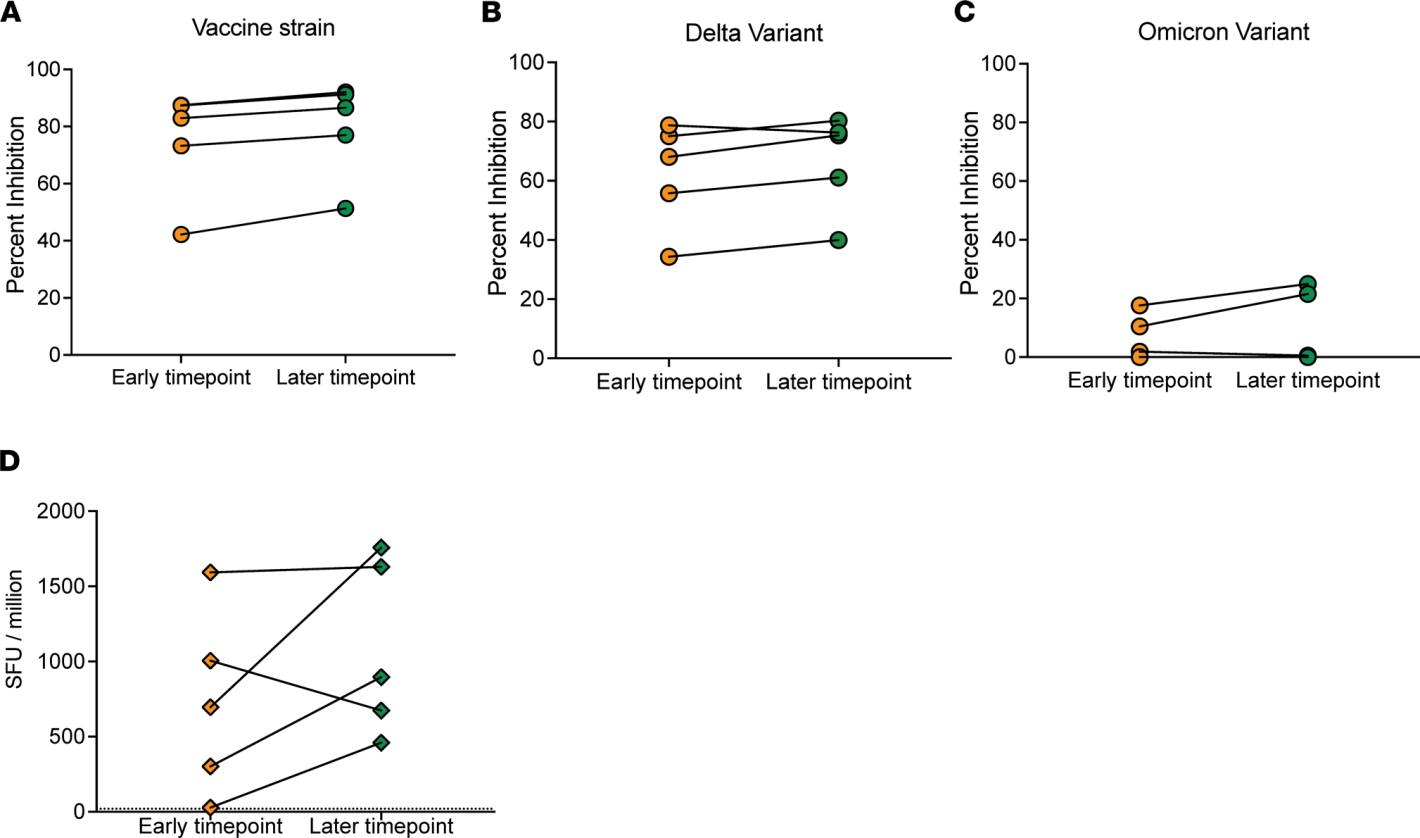
Characterization of longitudinal SARS-CoV-2–specific antibody and T cell responses in boosted vaccine recipients with breakthrough infections. Levels of antibodies that inhibit ACE2/spike binding for (**A**) the vaccine strain and (**B**) Delta and (**C**) Omicron variants in 5 breakthrough VRs at either an early (days 1–4) or a later (days 4–10) time point. (**D**) T cell responses measured as IFN-γ spot-forming units (SFU) per million PBMCs to vaccine strain peptides at an early or later time point.

In the studies described above, we compared responses from breakthrough VRs at a median of 11 days after symptom onset with those from postboost VRs at a similar time point after vaccination. In order to estimate the contributions of anamnestic responses induced by the breakthrough infection to the total responses seen at the time point most VRs were studied, we obtained samples from 5 boosted breakthrough VRs at early (median, 3 days after the onset of symptoms; range, 1–4 days) and later (median, 8 days; range, 6–10 days) time points. We compared inhibition of ACE2 binding to the vaccine strain as well as Omicron and Delta at the 2 time points in these 5 VRs (Figure 4, A–C) and found no significant increase in the level of inhibiting antibodies. We also found no significant difference in the T cell response to the vaccine strain spike peptides in this time frame (Figure 4D). In order to determine change in antibody levels after breakthrough infection, we compared levels 1–3 weeks and 1–3 months after breakthrough to levels found 1–3 weeks after boosting in VR21, VR26, VR37, and VR97. There was no appreciable decay in the levels of spike-binding antibodies (Supplemental Figure 3A). However, there was a modest decay in the inhibition of ACE2 binding to all 5 spike proteins in VR21, VR26, VR37, and VR97 between the postboost and 1- to 3-week breakthrough time point, followed by a subsequent increase at the 4- to 7-week time point (Supplemental Figure 3B).

## Discussion

Our study demonstrates robust antibody and T cell responses following the third booster dose in 4 VRs who went on to develop a breakthrough infection at a median of 45 days after the third vaccine dose. At 1–3 weeks after symptom onset, the breakthrough VRs had antibody levels against the vaccine strain spike protein that were comparable to those seen in postboost VRs at a similar time point after the third vaccine dose. Interestingly, the T cell responses to the vaccine strain spike S1 subunit were more robust in breakthrough VRs than in postboost VRs at this time point. Some important limitations of our study are the relatively small number of breakthrough participants studied, the semiquantitative nature of the Euroimmun assay, and the fact that we were not able to provide sequence confirmation of infection with the Omicron variant. However, taken together our data suggest that breakthrough infection can occur despite potent adaptive immune responses to the vaccine strain spike protein, even in young healthy boosted VRs. This is in contrast to studies that found lower neutralizing antibody and T cell responses in breakthrough VRs who were infected with the Alpha variant (20–22) and confirms a study showing high titers of spike-binding antibodies in patients with Omicron breakthrough infections (28). While only 4.4% of the participants in that study had received 3 vaccine doses, all the VRs in our study had received a booster shot. The difference in the ability of different variants to cause breakthrough infections in the face of robust vaccine-elicited immune responses may be due to the fact that the Omicron variant is more infectious than prior variants (2) and the fact that the heavily mutated spike protein of this variant is able to evade vaccine-elicited neutralizing antibody responses (3–8). The finding that prebreakthrough plasma from VR21, VR26, VR37, and VR97 mediated strong inhibition of ACE2/spike binding with the vaccine strain isolate but weaker inhibition with the Omicron variant may suggest that boosted VRs with narrow antibody responses to the spike protein may be more susceptible to breakthrough infections with VOCs. However, as seen in other studies (9–15), potent Omicron-specific T cell responses were present in all boosted VRs, and this may explain why breakthrough infections caused only mild symptoms in these individuals. It is interesting that infection appeared to enhance the T cell response but not the ACE2-inhibiting antibody response to the Omicron variant during the first 2 weeks after symptom onset. These findings will need to be confirmed in larger cohort studies, but they may potentially be due to a difference in epitope recognition by B and T cells. The Omicron variant effectively evades neutralizing antibodies but is recognized by T cells. Thus, the enhanced T cell response to Omicron variants may be due to both preexisting cross-reactive memory T cells and newly generated T cell responses specific for Omicron variant epitopes, whereas the antibody response would consist mainly of newly generated antibodies against the Omicron variant. Further studies will be needed to compare the ability of B and T cells to elicit variant-specific responses after initial priming with vaccine strain spike proteins. In summary, our data suggest that mild breakthrough infections with the Omicron variant can occur despite robust responses to mRNA booster vaccine. However, these vaccine-elicited responses do appear to protect against severe disease (16–19).

## Methods

### Study participants.

Study participants who experienced breakthrough infections after full vaccination followed by an additional booster dose are referred to herein as breakthrough VRs. The 18 breakthrough VRs had no comorbidities and a mean age of 30 years (range, 23–62 years). Fourteen of these VRs were female and 4 were male. COVID-19 diagnosis was made by PCR on sputum or nasal swab specimens in 14 participants and by an antigen test in 4 participants. All infected participants experienced mainly mild upper respiratory tract symptoms.

Fourteen of the breakthrough VRs received 3 doses of the BNT162b2 (Pfizer) vaccine, 3 received an initial dose of the Ad26.COV2 (Johnson & Johnson) vaccine followed by a booster dose of the mRNA1273 (Moderna) vaccine, and 1 received the 3 doses of the mRNA1273 vaccine (Table 1). The median time between the receipt of the booster dose and the onset of symptoms was 50 days (range, 14–92 days) for all 18 VRs. The median time between symptom onset and sampling for immune response analysis was 11 days (range, 6–19 days). As part of a separate analysis, we also obtained samples from 5 boosted VRs at early (median, 3 days after the onset of symptoms; range, 1–4 days) and later (median, 8 days; range, 6–10 days) time points. Furthermore, we tested preinfection immune responses in 4 boosted VRs (VR21, VR26, VR37, and VR97) between 5 and 21 days after the booster vaccine dose. The median time to symptom onset for these 4 participants was 45 days (range, 32–76 days). More information on breakthrough VRs is presented in Table 1.

Breakthrough VRs were compared with boosted study participants with no prior history of COVID-19 infections (i.e., postboost VRs). For postboost VRs, the first cohort was sampled 1–3 weeks following booster shots (*n =* 31). Fifteen of 31 of these participants were female. Twenty-eight participants received 3 doses of BNT162b2 vaccine, 1 received 3 doses of mRNA1273 vaccine, and 2 received 2 doses of BNT162b2 vaccine followed by a mRNA1273 booster dose. Age of study participants ranged from 21 to 60 years, with 10 participants aged 21–30 years, 7 participants aged 21–40 years, 7 participants aged 41–50 years, and the remaining 7 participants aged 51–60 years.

A second cohort of postboost VRs were sampled 1–3 months following booster doses (*n =* 13) and had no prior history of COVID-19 infections. Seven of 13 were female. Eleven received 3 doses BNT162b2 vaccinations, 1 received 3 doses of mRNA1273, and 1 received 2 doses of BNT162b2 followed by an mRNA1273 booster shot. Age ranged from 21–60 years, with 7 participants aged 21–30 years, 2 participants aged 31–40 years, 2 participants aged 41–50 years, and 2 aged 51–60 years.

A third cohort of postboost VRs were sampled 1–4 weeks following booster doses and were used for T cell response comparisons for the Omicron variant (*n =* 11). Six of 11 were female. All participants received 3 doses of BNT162b2. Age ranged from 21 to 60 years, with 4 aged 21–30 years, 2 aged 31–40 years, 4 aged 41–50 years, and 1 aged 51–60 years.

A cohort of individuals who received full doses of vaccination with no prior history of COVID-19 infections was sampled more than 6 months following their second dose of vaccination (i.e., preboost VRs, *n =* 21) and was also used for comparisons. Ten of 21 donors were female. Twenty received 2 doses of BNT162b2, and 1 received mRNA1273. Age ranged from 21 to 60 years, with 7 aged 21–30 years, 5 aged 31–40 years, 5 aged 41–50 years, and 4 aged 51–60 years.

### Spike-binding antibody assay.

The Euroimmun Anti-SARS-CoV-2 (IgG) ELISA assay was used to measure the titer of binding antibody against the vaccine-strain spike protein as previously described (25). Antibodies against the nucleocapsid protein were measured with the Bio-Rad Platelia SARS-CoV-2 Total Ab assay and used to rule out asymptomatic infection. Seven breakthrough VRs had positive responses at the time of breakthrough, and 2 others had indeterminate responses (Supplemental Figure 3C). Interestingly, 16 of 17 breakthrough VRs had detectable T cell responses to the nucleocapsid peptide pool at this time point (Supplemental Figure 3D).

### Receptor binding domain binding assay.

Antibodies against the receptor binding domain of SARS-CoV-2 were measured in plasma using the Meso Scale Diagnostics (MSD) Coronavirus Panel 3 IgG kit at a dilution of 1:5000, according to the manufacture’s protocols. This is an electrochemiluminescence sandwich ELISA-based assay used in multiple studies of SARS-CoV-2 antibodies (26). Each sample was measured in duplicate. Plates were read on a MESO QuickPlex SQ 120, and arbitrary units were calculated using the MSD Discovery Workbench software according to the manufacturer’s protocols. Conversion to WHO binding antibody units was performed by multiplying arbitrary units by the manufacturer’s verified conversion factor. Seropositivity cutoffs for SARS-CoV-2–specific antibodies were provided by the manufacturer and are based on convalescent samples. Data are presented on a log scale.

### ACE2/spike inhibition assay.

The MSD ACE2 inhibition assay measures the ability of plasma to inhibit ACE2 binding to full-length spike protein, a surrogate measure of neutralization. Previous data have indicated that a cutoff of 20% ACE2 inhibition is associated with measurable live virus–neutralizing antibody, including versus VOCs (26). Briefly, plasma from study participants was thawed, and ACE2 inhibition was measured using the ACE2 MSD V-PLEX SARS-CoV-2 23 kits according to the manufacturers’ protocols at a dilution of 1:100. Specifically, plates were precoated by the manufacturer with spike proteins corresponding to variants of interest (i.e., expressing key mutations). The plates were washed and incubated with plasma for 1 hour followed by the addition of human ACE2 protein conjugated with a SULFO-TAG (light-emitting label) for another hour. The plates were then washed, read buffer was added, and the plates were read with a MESO QuickPlex SQ 120 instrument per the manufacturer’s instructions. If the plasma fully bound the coated spike protein and blocked binding of the added ACE2, then no light was emitted during the electrical stimulation phase of the assay, corresponding to 100% ACE2 inhibition (full surrogate neutralization). Whereas, if there was no effective binding of spike by plasma, then the SULFO-TAG ACE2 fully bound the coated spike protein and illuminated during activation of the chemiluminescent plate, corresponding to 0% inhibition. At least 4 wells were left blank for calibration to 0% inhibition. Results were reported as a percentage of ACE2 inhibition based on the equation provided by the manufacturer (1 – average sample ECL/average ECL signal of blank well) × 100, where ECL stands for electrochemiluminescence.

### ELISpot assay.

The IFN-γ ELISpot assay was used to analyze T cell responses to SARS-CoV-2 spike and nucleocapsid peptide pools (BEI Resources) and the S1 subunit of the vaccine strain and Omicron variant spike proteins (Genscript Biotech Corporation). Patient PBMCs were incubated with peptides for 20 to 24 hours or with the spike proteins for 36 to 40 hours before the plates were developed as previously described (29).

### Statistics.

Statistical analyses were performed with GraphPad Prism 9.2.0. Statistical tests performed are provided in figure legends. If unpaired, statistical comparisons were done using ordinary 1-way ANOVA and Holm-Šidák’s multiple-comparison test, with a single pool variance used. Paired analyses were done using repeated-measures 1-way ANOVA with Geisser-Greenhouse corrections and Šidák’s multiple-comparison test, with individual variances computed for each comparison. *P* values of less than 0.05 were considered significant.

### Study approval.

The study was approved by the IRB of Johns Hopkins University. Written informed consent was obtained from all study participants prior to their inclusion in the study.

## Author contributions

BAW, CCG, TYA, TSJ, and EJB performed experiments. AGD, NF, DAW, AHY, and AID contributed reagents. OL, ALC, HHM, AHK, and JNB supervised experiments.

## Supplementary Material

Supplemental data

ICMJE disclosure forms

## Figures and Tables

**Table 1:**
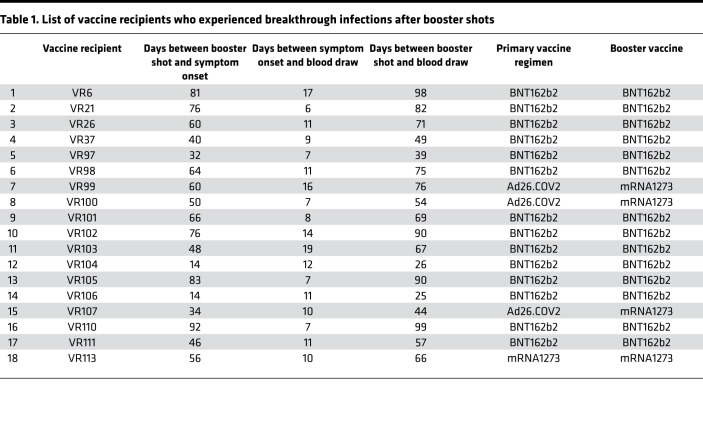
List of vaccine recipients who experienced breakthrough infections after booster shots

## Version 1 04/07/2022

In-Press Preview

## Version 2 05/23/2022

Electronic publication

## Version 3 02/23/2024

The National Cancer Institute grant number was updated
