# Supplementary material for: SARS-CoV-2–specific immune responses in boosted vaccine recipients with breakthrough infections during the Omicron variant surge
Source: JCI Insight. 2022 May 23;7(10):e159474. doi: 10.1172/jci.insight.159474 (PMC9220829; doi:10.1172/jci.insight.159474)
Supplement: Supplemental data [file jciinsight-7-159474-s006.pdf]

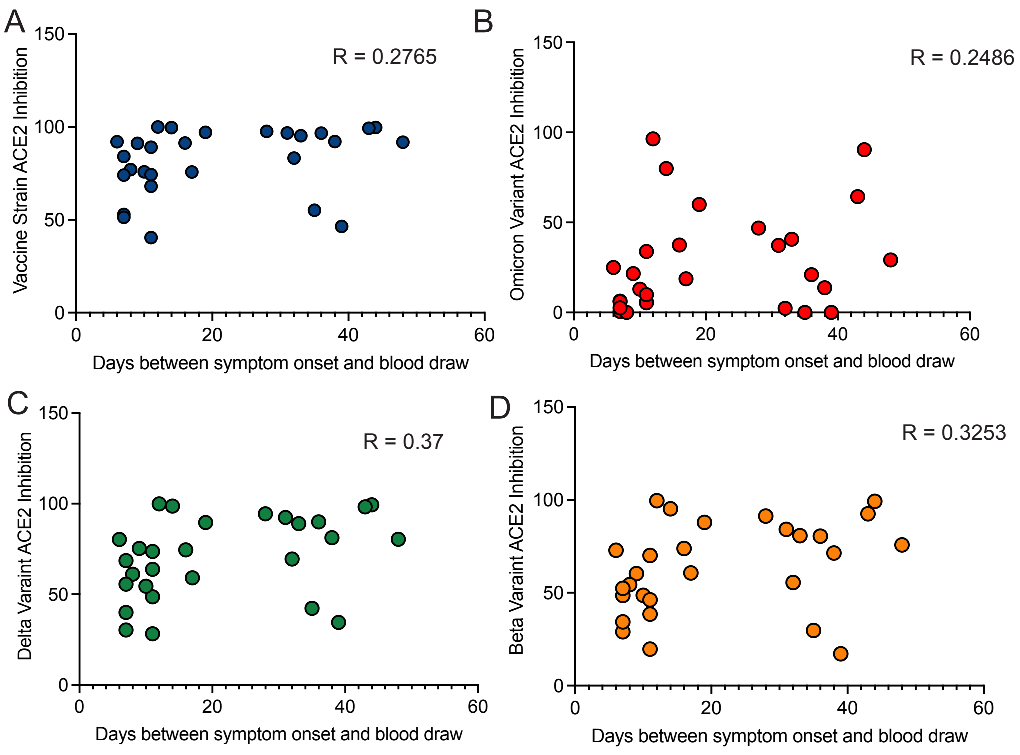

Supplemental Figure 1 Correlation of ACE2 inhibiting antibodies to days since symptom onset. ACE2 inhibiting antibodies to vaccine strain (A), omicron (B), Delta (C) and Beta (D) for breakthrough VRs were correlated with days between symptom onset and blood draw. Pearson correlation coeficcients were computed and R values are displayed on each graph.

Supplemental FIgure 2

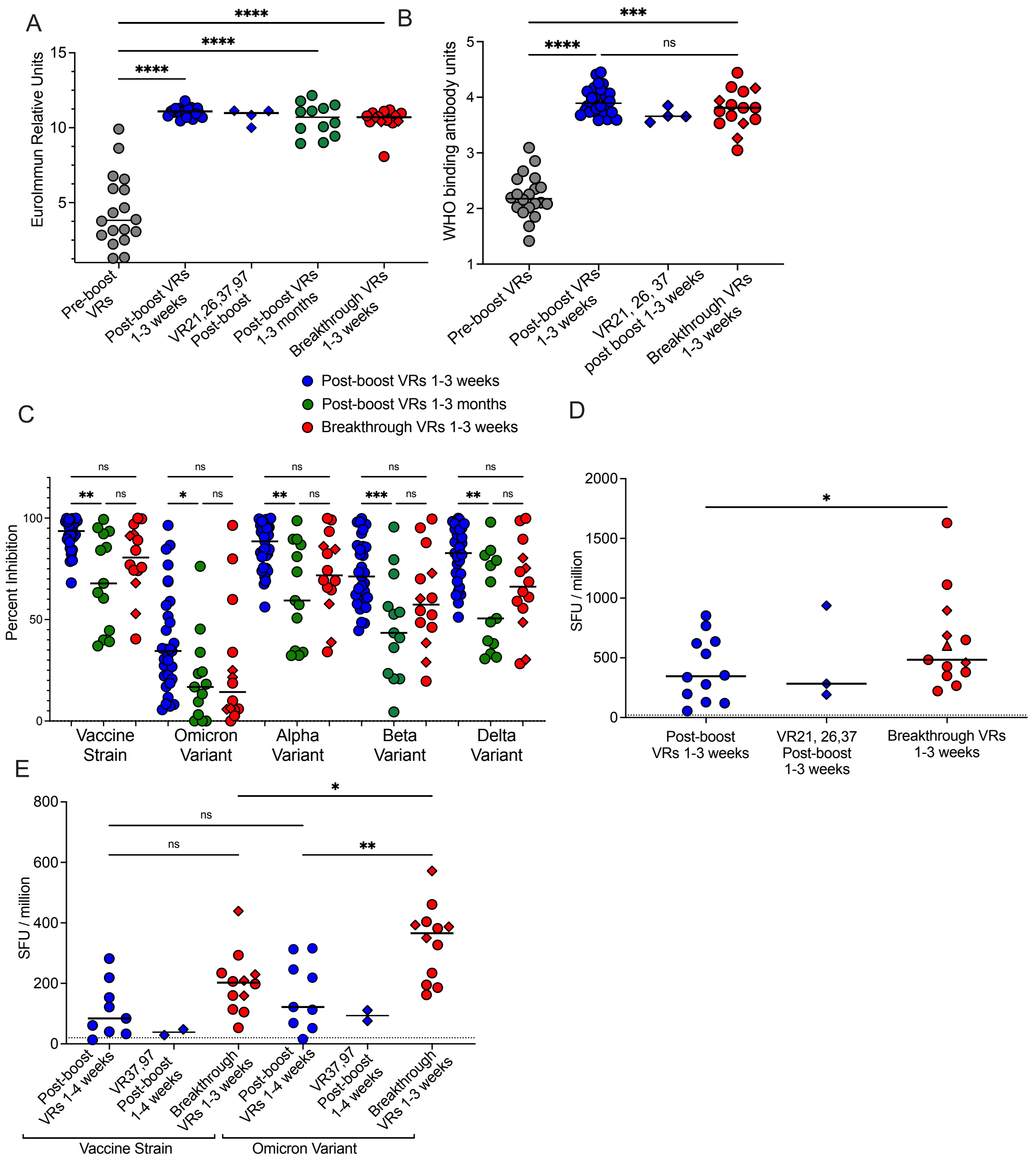

Supplemental Figure 2: Characterization of immune responses following breakthrough infections in breakthrough VRs who only received mRNA vaccinations. Data from Figure 2 and 3 are displayed with breakthrough donors receiving Ad26.COV2 (Johnson & Johnson) excluded. (A) Spike binding antibodies found in mRNA vaccinated individuals prior to their booster shots (Pre-boost VRs), individuals 1-3 weeks (Post-boost VRs 1-3 weeks) or 1-3 months (Post-boost 1-3 months) after their booster shot, breakthrough VRs 1-3 weeks after symptom onset, and VR21, VR26, VR37 and VR 97 1-3 weeks after their booster shots and before they experienced breakthrough infections. The orange diamonds represent VR 21, 26, 37 and 97 at the breakthrough time point. (B) Receptor binding domain (RBD) antibodies in Pre-boost, Post-boost, and Breakthrough VRs as well as VR21, VR26, VR37 at the post-boost time point and prior to infection. The orange diamonds represent VR26 and 37 at the breakthrough time point. (C) Levels of antibodies that inhibit ACE2/spike binding in VRs in Post-boost VRs 1-3 weeks or 1-3 months after their booster shot, and breakthrough VRs 1-3 weeks after symptom onset. The orange diamonds represent VR21, VR26, VR37, and VR 97. (D) IFN- ELIspot assay to vaccine strain overlapping spike peptide pools and (E) IFN- ELIspot assay to S1 protein from vaccine strain or omicron variant in post-boost VRs, in VR21, VR26, and VR37 1-3 weeks post-boost, and in breakthrough VRs.IFN-γ spot forming units (SFU) per million PBMCs are shown. Orange diamonds represent VR21, 26, 37 and 97 at the breakthrough time point. Statistical comparisons were done using Ordinary one-way ANOVA (unpaired) and Holm-Sidak's mulitiple comparison test, with a single pool variance used. *(0.0332), **(0.0021), ***(0.0002), **** (<0.0001).

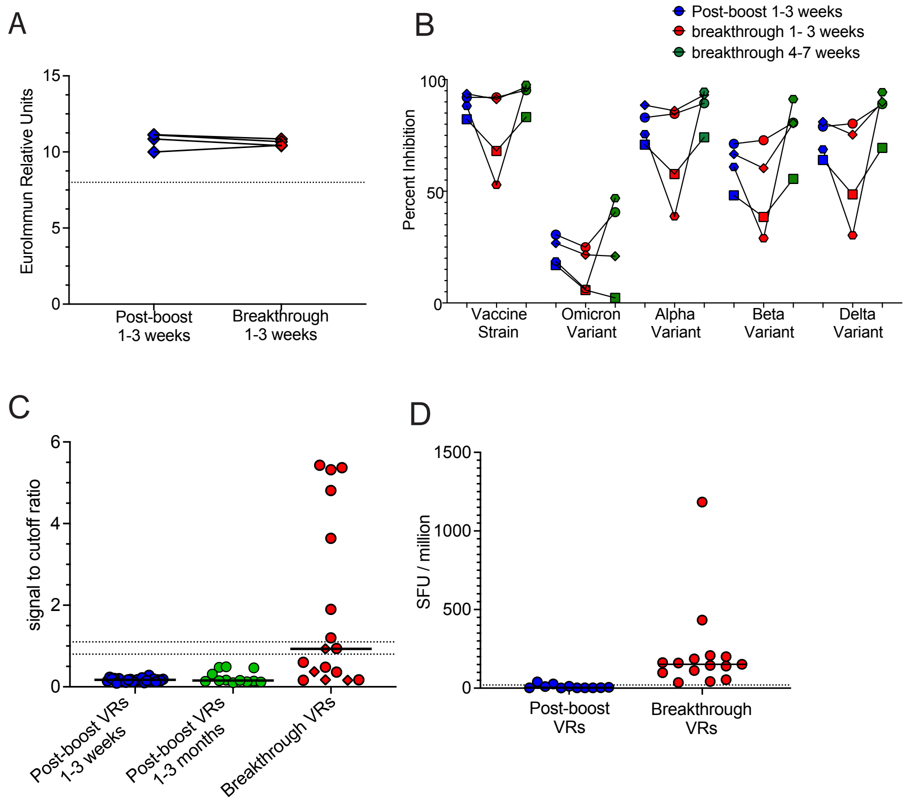

Supplementary Figure 3: Antibody and T cell responses to spike and nucleocapsid proteins. (A) Spike binding antibodies present in VR21, VR26, VR37 and VR97 after booster shots and after breakthrough infection. (B) Levels of antibodies inhibiting ACE2/spike binding with the vaccine strain and with variants of concern in VR21, VR26, VR37 and VR97 after booster shots and after breakthrough infection. (C) Antibody responses to the nucleocapsid protein in post-boost VRs and breakthrough VRs. The lower line represents cutoff for negative responses and the upper line denotes the cutoff for indeterminate responses. (D) T cell responses to nucleocapsid peptides in VRs after the booster shot and in VRs with breakthrough infection.
